# IgG4-related fibrosing mediastinitis diagnosed with computed tomography-guided percutaneous needle biopsy: Two case reports and a review of the literature

**DOI:** 10.1097/MD.0000000000010935

**Published:** 2018-06-01

**Authors:** Satoshi Takanashi, Mitsuhiro Akiyama, Katsuya Suzuki, Kotaro Otomo, Tsutomu Takeuchi

**Affiliations:** Division of Rheumatology, Department of Internal Medicine, Keio University School of Medicine, Tokyo, Japan.

**Keywords:** computed tomography guided percutaneous needle biopsy, fibrosing mediastinitis, IgG4-related disease, M2 macrophage, storiform fibrosis

## Abstract

**Rationale:**

Immunoglobulin G4-related disease (IgG4-RD) is a fibroinflammatory disease characterized by elevated serum IgG4 levels with infiltration of IgG4+ plasma cells and severe fibrosis in affected tissues. Recently, idiopathic fibrosing mediastinitis (FM), an extremely rare fibroinflammatory disorder, has been recognized as a form of IgG4-RD. As IgG4-RD can be treated by glucocorticoids, identification of the etiology of FM by surgical biopsy is essential; however, mediastinal biopsy is often difficult. We report 2 cases of IgG4-related FM successfully diagnosed with computed tomography (CT)-guided percutaneous needle biopsy.

**Patient concerns:**

Case 1 was a 66-year-old woman with elevated serum C-reactive protein without any symptoms and case 2 was 78-year-old woman with abnormal mediastinal contour on chest x-ray. By further work-up, both cases were found to have mediastinitis accompanied by elevated serum IgG4. CT-guided percutaneous needle biopsy revealed massive infiltration of IgG4+plasma cells along with storiform fibrosis.

**Diagnosis:**

IgG4-related FM.

**Interventions:**

Glucocorticoid therapy.

**Outcome:**

The treatment resulted in significant improvement of the lesions after 3 months.

**Lessons:**

Early recognition and diagnosis of IgG4-related FM is essential because a delay in appropriate treatment initiation leads to progressive fibrosis with irreversible organ damage and poor prognosis. Our cases highlight CT-guided percutaneous needle biopsy as a promising option for histological examination in patients with IgG4-related FM.

## 1 Introduction

Immunoglobulin G4-related disease (IgG4-RD) is a fibroinflammatory disease characterized by elevated serum IgG4 levels, infiltration of IgG4^+^ plasma cells, and severe fibrosis in the affected tissues.^[1]^ IgG4-RD frequently involves the pancreas, lacrimal glands, salivary glands, bile duct, kidneys, lungs, and retroperitoneum, but can potentially affect any organ.^[1]^

Fibrosing mediastinitis (FM) is one of the rare fibroinflammatory diseases that affects the mediastinum.^[2,3]^ FM can be classified as idiopathic or secondary based on its etiology,^[2]^ and identification of the etiology is clinically important for selecting the correct treatment. Delay in diagnosis and initiation of appropriate treatment for FM results in irreversible organ damage, which can lead to severe disability and poor prognosis.^[2,3]^

Recently, IgG4-RD has been linked to some cases of “idiopathic” FM, which is now called “IgG4-related FM.”^[2]^ As IgG4-RD is treatable with glucocorticoids,^[1]^ clinicians should recognize FM and perform a biopsy to identify the etiology. However, surgical biopsy in patients with FM is often difficult if a lesion's location is paravertebral or adjacent to vital organs. Additionally, FM usually affects the elderly, who often have comorbidities.

We report 2 cases of IgG4-related FM successfully diagnosed with low-invasive computed-tomography (CT)-guided percutaneous needle biopsy.

Ethical approval was waived according to the regulations in Japan. Informed consent were obtained from the patients.

## 2 Case presentations

### 2.1 Case 1

**Figure 1:**
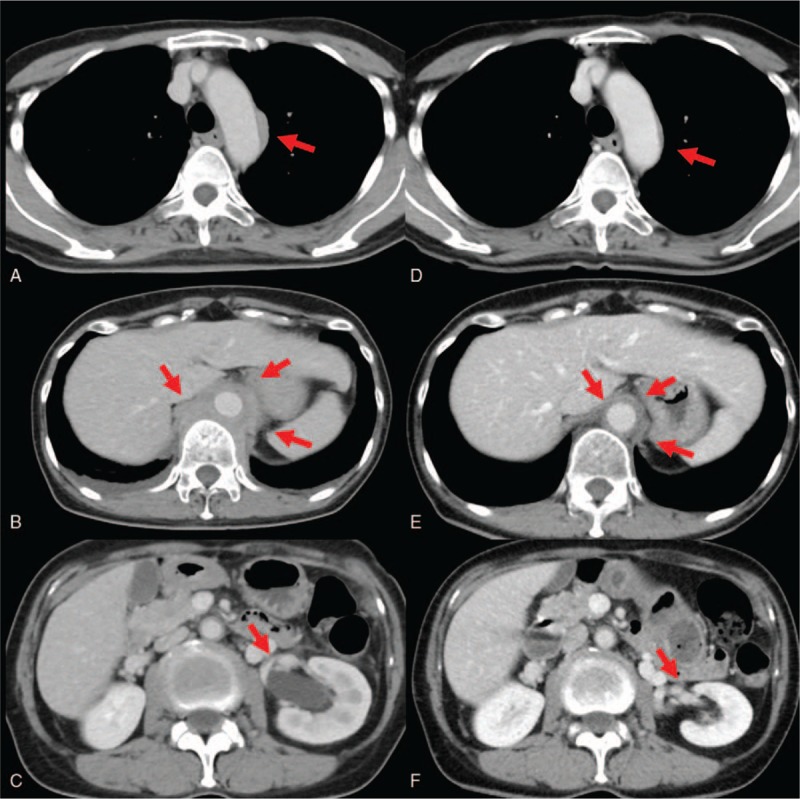
Radiological findings of case 1. A: Soft tissue mass on aortic arch before therapy. B: Soft tissue mass on descending thoracic aorta before therapy. C: Left hydronephrosis before therapy. D: Soft tissue mass on aortic arch 3 months after therapy. E: Soft tissue mass on descending thoracic aorta 3 months after therapy. F: Hydronephrosis 3 months after therapy.

**Figure 2:**
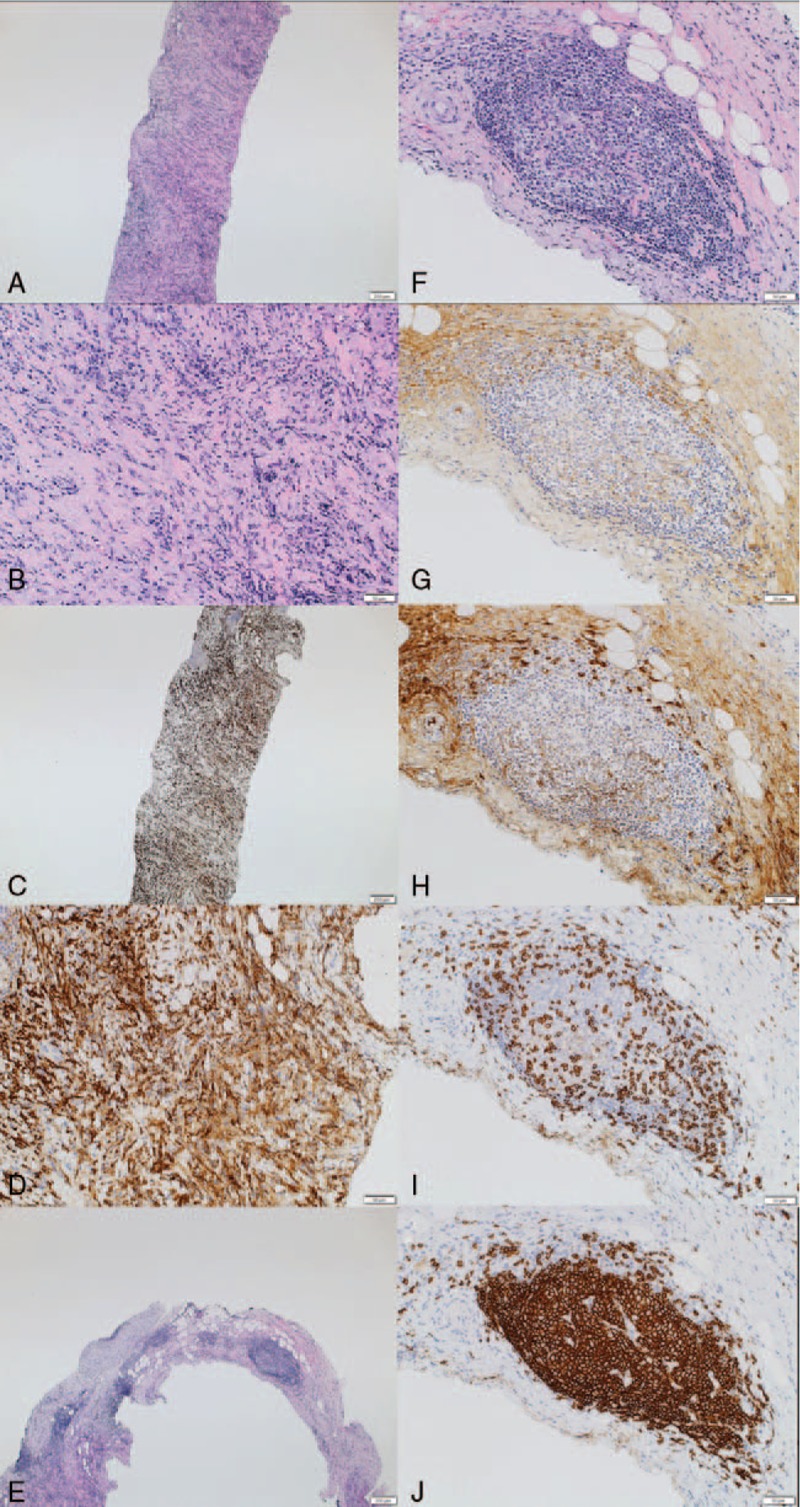
Histological findings of paravertebral mass in case 1. A: Hematoxylin and eosin staining, low power field; dense lymphoplasmacytic infiltration, and storiform fibrosis were observed. B: Hematoxylin and eosin staining, high power field. C: CD163 staining, low power field; massive infiltration of CD163^+^ M2 macrophages were observed. D: CD163 staining, high power field. E: Hematoxylin and eosin staining, low power field; hyperplastic ectopic germinal center formation was observed. F: Hematoxylin and eosin staining, high power field. G: IgG staining. H: IgG4 staining. I: CD3 staining. J: CD20 staining.

A 66-year-old woman with a history of type 2 diabetes mellitus presented with elevated serum C-reactive protein (CRP) (6.15 mg/dL, normal range <0.35 mg/dL) at a regular checkup without any symptoms in June 2017. CT findings revealed a soft tissue mass involving the aortic root, aortic arch, descending thoracic and abdominal aorta, and left iliac artery. Paraaortic mass lesions were identified adjacent to the thoracic spine. She was admitted to our hospital for further workup. Physical examination revealed a normal blood pressure of 138/88 mmHg and body temperature of 36.6 °C. The findings of ocular, face, neck, lungs, cardiovascular, abdominal, neurological, and skin examinations were normal. Laboratory tests showed elevated serum IgG (2004 mg/dL, normal range: 870–1700 mg/dL), IgG4 (276 mg/dL, normal range: 4.8–105 mg/dL), and soluble interleukin-2 receptor (sIL-2R; 502 U/mL, normal range: 142–500 U/mL). Serum immunoglobulin E (IgE) level was within the normal range. Other blood tests including blood count, serum electrolytes, liver enzyme levels, and serum creatinine were within the normal range. The enzyme-linked tuberculosis immunospot assay (ELISPOT) T-SPOT.*TB* (Oxford Immunotec, Oxford, UK), antinuclear antibody (ANA), rheumatoid factor (RF), and anti-neutrophil cytoplasmic antibody (ANCA) were negative. C3 and C4 were within the normal range. Serum interleukin-6 (IL-6) level was elevated (5.4 pg/mL, normal range 0–4.0 pg/mL). Blood cultures did not identify any pathogens. Antibodies to syphilis were negative. Urinalysis showed no proteinuria, hematuria, white blood cells, or casts. Chest x-ray revealed small pleural effusions. A contrast-enhanced CT demonstrated interval enlargement of the mass around the aorta and development of left hydronephrosis (Fig. 1A–C). IgG4-related FM/retroperitoneal fibrosis was suspected. We performed CT-guided percutaneous needle biopsy of the paravertebral mass. Histological findings showed dense lymphoplasmacytic infiltration along with storiform fibrosis (Fig. 2A and B). Massive infiltration of CD163^+^ M2 macrophages in the fibrotic lesions (Fig. 2C and D) and hyperplastic ectopic germinal center formation (Fig. 2E and F) were observed. Immunohistochemical staining showed that >40% of plasma cells with IgG immunoreactivity (Fig. 2G) were positively immunolabeled with the IgG4 antibody (Fig. 2H). The ectopic germinal centers consisted of CD3^+^ T cells (Fig. 2I) and CD20^+^ B cells (Fig. 2J). We diagnosed IgG4-related FM/retroperitoneal fibrosis based on the 2011 comprehensive diagnostic criteria.^[4]^ She received 30 mg/d (0.6 mg/kg) of prednisolone (PSL) as induction therapy. Kidney ultrasound performed 14 days after initiation of therapy revealed improvement of the left hydronephrosis. After 3 months, the levels of serum IgG4 and CRP had decreased to 78 and 0.06 mg/dL, respectively. CT findings also revealed remarkable improvement of the mass around the thoracic aorta and of the hydronephorosis (Fig. 1D–F). The dose of PSL was gradually tapered. There was no recurrence over 6 months.

### 2.2 Case 2

**Figure 3:**
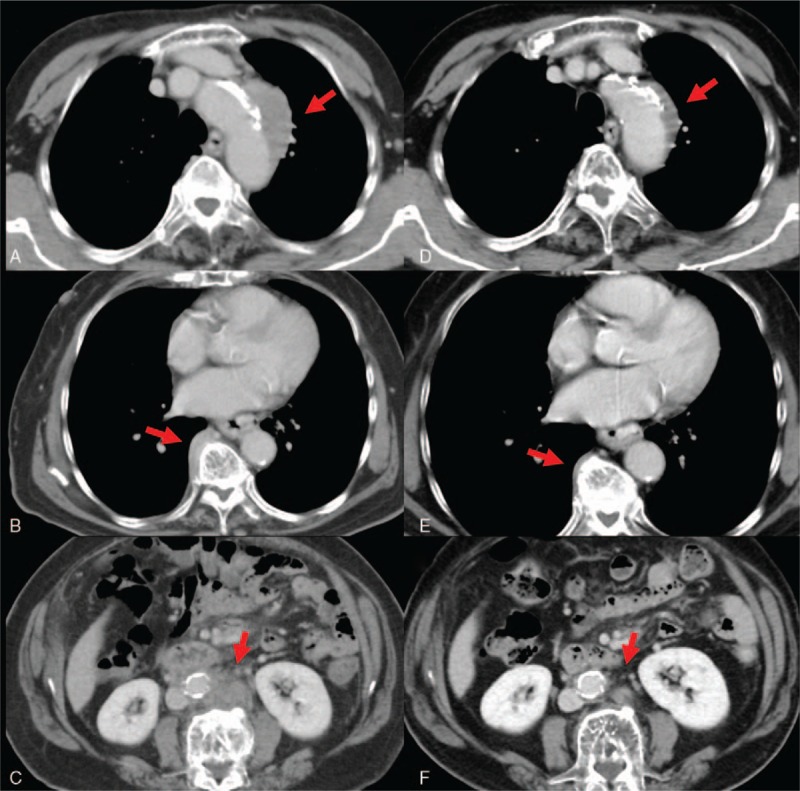
Radiologic findings of case 2. A: Soft tissue mass on aortic arch before therapy. B: Thoracic paravertebral soft tissue mass before therapy. C: Retroperitoneal fibrosis before therapy. D: Soft tissue mass on aortic arch 3 months after therapy. E: Paravertebral mass 3 months after therapy. F: Retroperitoneal fibrosis 3 months after therapy.

**Figure 4:**
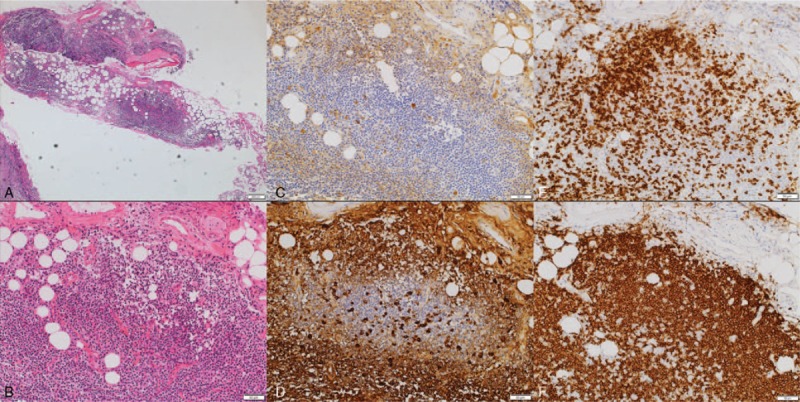
Histological findings of paravertebral mass in case 2. A: Hematoxylin and eosin staining, low power field; dense lymphoplasmacytic infiltration, storiform fibrosis, and hyperplastic ectopic germinal center formation were observed. B: Hematoxylin and eosin staining, high power field. C: IgG staining. D: IgG4 staining. E: CD3 staining. F: CD20 staining.

A 78-year-old woman with a history of type 2 diabetes mellitus, fatty liver, hypertension, and cholecystitis was found to have an abnormal mediastinal contour on chest x-ray on a routine health checkup in November 2016. She visited another hospital and her laboratory data showed elevated serum IgG (4164 mg/dL) and IgG4 (1170 mg/dL). CT findings revealed soft tissue masses involving the aortic arch, abdominal aorta, and perivertebral thoracic soft tissues (Fig. 3A–C). Retroperitoneal, mediastinal, paraaortic, and pelvic lymphadenopathy were also found. She was admitted to our hospital for further workup in April 2017. Physical examination revealed a blood pressure of 137/90 mmHg and body temperature of 36.6 °C. Laboratory tests revealed elevated serum IgG (3685 mg/dL), IgG4 (1940 mg/dL), IgE (290 IU/mL), and sIL-2R (1061 U/mL). Other blood tests, including blood count, serum electrolytes, serum creatinine, and CRP, were within the normal range. Serum liver enzymes were slightly elevated, possibly due to her fatty liver: aspartate aminotransferase (54 U/L, normal range 10–35 U/L), alanine aminotransferase (45 U/L, normal range 5–40 U/L), alkaline phosphatase (391 U/L, normal range 100–320 U/L), and γ-GTP (42 U/L, normal range 5–40 U/L). The ANA titer was 1:160 (homogeneous, speckled pattern) and RF was 295 IU/mL (normal range: 0–15 IU/mL). ANCA, anti-dsDNA antibody, anti-SS-A antibody, anti-SS-B antibody, and anti-cyclic citrullinated peptide antibody were all negative. C4 was 7 mg/dL (normal range, 13–35 mg/dL), and C3 was within the normal range. Blood cultures and T-SPOT.*TB* were negative. Urinalysis showed no proteinuria, hematuria, white blood cells, or casts. To confirm the diagnosis, we performed a CT-guided percutaneous needle biopsy of a paravertebral mass. Histological findings revealed dense lymphoplasmacytic infiltration with storiform fibrosis and hyperplastic ectopic germinal center formation (Fig. 4A and B). Immunohistochemical staining showed that 50% of plasma cells with IgG immunoreactivity (Fig. 4C) were positively immunolabeled with the IgG4 antibody (Fig. 4D). The ectopic germinal centers consisted of CD3^+^ T cells (Fig. 4E) and CD20^+^ B cells (Fig. 4F). We diagnosed IgG4-related FM/retroperitoneal fibrosis based on the 2011 comprehensive diagnostic criteria.^[4]^ She received 30 mg/d of PSL as induction therapy and 3 months later we performed a follow-up CT that revealed marked improvement of the paravertebral mass/retroperitoneal fibrosis (Fig. 3D–F). Serum IgG and IgG4 had decreased to 1310 and 290 mg/dL, respectively. The dose of PSL was tapered and there was no recurrence over 8 months of follow-up.

## 3 Discussion

We described 2 cases of IgG4-related FM diagnosed with CT-guided percutaneous needle biopsy. Both cases showed the typical radiological features of FM, histological findings for IgG4-RD, high serum IgG4, and response to glucocorticoid therapy.

Our cases are noteworthy for several reasons. First, considering that delayed diagnosis and initiation of early appropriate treatment for FM results in irreversible organ damage and potential subsequent severe disability and poor prognosis,^[2,3]^ and that IgG4-related FM is treatable by glucocorticoid therapy, clinicians should recognize IgG4-related FM and perform a biopsy to identify the etiology of FM. Second, CT-guided percutaneous needle biopsy is one option for the pathological diagnosis of IgG4-related FM that is less invasive than surgical biopsy for accessing the mediastinum. Finally, we found hyperplastic ectopic germinal centers consisting of T and B cells and massive infiltration of M2 macrophages, along with storiform fibrosis, in the affected tissues of IgG4-related FM. This suggests that T–B interaction and M2 macrophages may be involved in the pathogenesis of IgG4-related FM, as commonly reported in other sites of IgG4-RD.^[5–10]^

FM is a rare fibroinflammatory disease.^[2,3]^ It can be asymptomatic and found incidentally on radiological examination, or symptoms such as dyspnea, hemoptysis, dysphagia, and chest pain can occur in cases where the FM mass compresses adjacent organs (blood vessels, airway, esophagus, and heart).^[2,3]^ FM can be classified as idiopathic or secondary based on its etiology.^[2,3]^ Secondary FM can follow infections (*Histoplasma capsulatum*, aspergillosis, cryptococcosis, tuberculosis, atypical mycobacterial infections, and nocardiosis), malignancies (particularly lymphoma and mesothelioma), autoimmune diseases (anti-neutrophil cytoplasmic antibody-associated vasculitis and Behçet disease), and sarcoidosis. It can also develop in response to radiation therapy and drugs (methysergide maleate).^[2]^ If no clear cause is identified, the disease can be classified as idiopathic. Recent reports suggest that “idiopathic” FM in some patients can be attributed to IgG4-RD.^[2,11–19]^ We have described 2 cases of “idiopathic” FM that showed extensive IgG4 and IgG4^+^ plasma cell infiltration along with storiform fibrosis in affected tissues, eventually diagnosed as IgG4-related FM. Both cases were successfully treated with glucocorticoid therapy. Considering that a delay in diagnosis and treatment may cause severe disability and poor prognosis in FM patients,^[2,3]^ clinicians should consider IgG4-related FM in cases of mediastinal masses to allow early treatment.

We have reviewed all cases previously published as “IgG4-related FM” including our 2 cases (Table 1). A total of 15 cases were identified. Although age (median 56 years, range 31–83 years) and sex distribution (male to female ratio 11:4) tended to be similar to those for other manifestations of IgG4-RD, the majority of FM patients demonstrated elevated serum CRP (median 2.51 mg/dL, range 0.03–9.68 mg/dL). While IgG4-RD generally presents with normal CRP levels,^[20,21]^ some forms of IgG4-RD, such as IgG4-related retroperitoneal fibrosis, periaortitis, and inflammatory aortic aneurysms, can present with high serum CRP.^[22–24]^ In this regard, it should be noted that IgG4-related FM can also present with high serum CRP. While the underlying differences between IgG4-RD patients with normal and high CRP remain unclear, Kasashima et al^[23]^ recently reported excessive levels of local and serum IL-6 in patients with IgG4-related inflammatory aortic aneurysms, with alternatively activated (M2) macrophages in the adventitia producing IL-6. In line with these findings, case 1 in the present study showed high serum CRP as well as high serum IL-6. Therefore, high serum CRP in IgG4-related FM may be attributed to excessive local production of IL-6. In terms of other clinical features of published IgG4-related FM cases, the median level of serum IgG4 was 392 mg/dL (range 127–3300 mg/dL), and involvement included retroperitoneal fibrosis (7 cases, 47%), lymph nodes (2 cases, 13.3%), pancreas (1 case, 6.6%), lung (1 case, 6.6%), and dacryosialadenitis (1 case, 6.6%). Further accumulation of IgG4-related FM cases will help to elucidate the clinical features.

**Table 1:**
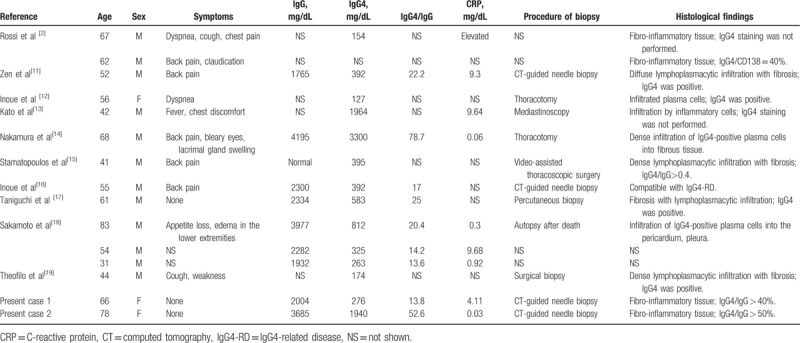
Literature review of cases with IgG4-related fibrosing mediastinitis.

While severe fibrosis is one of the important characteristics of IgG4-related FM, the mechanism underlying fibrosis formation is unclear. Recently, several studies reported that M2 macrophages contribute to fibrosis formation by producing CC-chemokine ligand-18 in IgG4-RD.^[5,6]^ Consistent with these findings, case 1 in the present study showed massive infiltration of CD163^+^ M2 macrophages in the area affected by storiform fibrosis associated with IgG4-related FM. This suggests that M2 macrophages are involved in the fibrosis process in IgG4-related FM.

Recent studies have suggested that follicular helper T cells are involved in ectopic germinal center formation and IgG4-secreting B-cell maturation and differentiation at sites affected by IgG4-RD.^[7–10]^ Both of our cases showed hyperplastic ectopic germinal center formation at affected sites (Figs. 2E–H and 4A–D). Furthermore, these ectopic germinal centers contained collections of T cells and B cells (Figs. 2I, J and 4E, F). Therefore, T–B interaction in the ectopic germinal center may play an important role in IgG4-secreting B-cell maturation and differentiation in IgG4-related FM.

The anatomical location of FM often makes it difficult for clinicians to perform a biopsy; however, because IgG4-RD is treatable by glucocorticoid therapy, it is important to first consider the histological diagnosis of IgG4-related FM and to exclude other diseases. In both of our cases, we successfully performed CT-guided percutaneous needle biopsy, and obtained specimens suitable for accurate diagnosis. Considering that IgG4-RD affects elderly patients with frequent comorbidities, and that CT-guided percutaneous biopsy is less invasive than surgical biopsy, CT-guided percutaneous needle biopsy is a useful option for obtaining tissue specimens for diagnosis of IgG4-related FM.

The prognosis of FM is affected by the enlargement of fibrous masses that compress adjacent organs.^[2,3]^ Our cases showed dramatic improvement of the lesions with glucocorticoid therapy. The long-term prognosis of IgG4-related FM remains unclear.

## 4 Conclusion

IgG4-related FM should be included in the differential diagnosis when clinicians find fibrosis in the mediastinum. It is important to screen for systemic organ involvement and to measure serum IgG4. CT-guided percutaneous needle biopsy is very useful and effective for an accurate diagnosis.

## Author contributions

**Conceptualization:** Satoshi Takanashi.

**Data curation:** Satoshi Takanashi.

**Writing – original draft:** Satoshi Takanashi.

**Writing – review and editing:** Satoshi Takanashi, Mitsuhiro Akiyama, Katsuya Suzuki, Kotaro Otomo, Tsutomu Takeuchi.
